# New Approach to a Practical Quartz Crystal Microbalance Sensor Utilizing an Inkjet Printing System

**DOI:** 10.3390/s141120468

**Published:** 2014-10-30

**Authors:** Yusuke Fuchiwaki, Masato Tanaka, Yoji Makita, Toshihiko Ooie

**Affiliations:** 1 Health Research Institute, National Institute of Advanced Industrial Science and Technology (AIST), 2217-14, Hayashi-cho, Takamatsu, Kagawa 761-0395, Japan; E-Mails: mst-tanaka@aist.go.jp (M.T.); y-makita@aist.go.jp (Y.M.); toshihiko-ooie@aist.go.jp (T.O.); 2 CEA-LETI, MINATEC, Campus, 17 rue des Martyrs, 38054 Grenoble Cedex 9, France

**Keywords:** QCM, inkjet print, inexpensive

## Abstract

The present work demonstrates a valuable approach to developing quartz crystal microbalance (QCM) sensor units inexpensively for reliable determination of analytes. This QCM sensor unit is constructed by inkjet printing equipment utilizing background noise removal techniques. Inkjet printing equipment was chosen as an alternative to an injection pump in conventional flow-mode systems to facilitate the commercial applicability of these practical devices. The results demonstrate minimization of fluctuations from external influences, determination of antigen-antibody interactions in an inkjet deposition, and quantification of C-reactive protein in the range of 50–1000 ng(x000B7)mL^−1^. We thus demonstrate a marketable application of an inexpensive and easily available QCM sensor system.

## 1. Introduction

The quartz crystal microbalance (QCM) is a versatile device in the field of physical, chemical, and biological sensors [1]. One unique advantage of QCM sensors is that they can be used as sensor devices without the need for labeling agents. As an online sensor, its working principle is based on the fact that changes in oscillation frequency are proportional to changes in the mass deposited on the electrode surface. Due to the higher sensitivity and lower detection limits of QCM sensors compared to traditional methods, many advances based on QCM sensors have been implemented, including chemical sensors based on RFID-Tags [2], sensors imprinted with yeast cells [3], direct-detection sensor systems in complex liquids [4], stable-detection enabling systems in continuous air flow [5], sensor devices using QCM-D [6], and practical prototype equipment combining amperometric measurements [7].

A higher oscillation frequency of the QCM sensor will lead to greater sensitivity. On the other hand, baseline signals are greatly amplified by external influences such as temperature fluctuations, vibrations, flow, and other factors. QCM sensor experiments are operated with a sequential multi-step procedure, for example, sample injection, blocking, and washing to remove excess sample from the bound analyte. Consequently, experiments are performed in flow-through mode because this approach can be automated and intermolecular recognition can be stably observed [8,9]. Although the utility of the flow-through mode has been amply demonstrated, a “batch-type” system remains an ideal QCM sensor. A “batch-type” sensor system has several practical advantages: (1) the sensor unit is available for use immediately; (2) construction is simple; (3) little maintenance is required; (4) operating costs are very low. However, for a “batch-type” system, several issues must be addressed, including the instability of background noise when the solution is changed to trigger the bound/free (B/F) states and a high level of technical expertise is required to operate batch-type sensors.

Recently, we succeeded in detecting the deposition of a picoliter amount of liquid by removing external fluctuations using both a 30 MHz QCM and a perforated stainless-steel cover [10]. The dispersion of the QCM frequency change per shot could be monitored and different concentrations of antibodies could be detected in an effective and highly reproducible manner. This proposed technique provided significantly decreased background noise caused by external sources such as air flow and temperature fluctuations, and allowed accurate determination of antibodies at a constant temperature.

The technology of inkjet printing is a critical component of microbioanalysis systems. Since the ability of inkjets to print 3D structures was realized, they have been used to deposit micro amounts of biomedical fluids [11–19]. Inkjet deposition can be performed in picoliter quantities in a single process with drop-on-demand (DOD) control.

Based on this experience and knowledge, in this study we investigated intermolecular recognition in real-time using a batch-type QCM sensor system, which consists of only commercially available devices. The method is based on the detection of a given change in the frequency generated by releasing nonspecific binding compounds from the QCM sensor surface right after an inkjet deposition of the surfactant aqueous solution. The nonspecific antigen is released by the deposition of an aqueous surfactant solution while the antigen specifically-bound to the antibody on the QCM sensor is not released. We attempted to detect this difference in real time in this study. This paper describes the development of a QCM sensor utilizing a piezoelectric inkjet printing technique to minimize fluctuations from external influences, facilitating an easily available batch-type QCM sensor system.

## 2. Experimental Section

### 2.1. Reagents and Materials

A human ***C-reactive protein (CRP)*** EIA Kit was purchased from CycLex Co., Ltd. (Nagano, Japan). CRP is a widely used biomarker for evaluating inflammation, and was employed here as a model antigen. Recombinant human adiponectin as a standard was purchased from R&D Systems Inc. (Minneapolis, MN, USA). Bovine serum albumin (BSA; 96%–99%) was obtained from Sigma Chemical Co. (Tokyo, Japan). All chemicals and solvents were of analytical grade and used as received. Standard stock solutions containing antigens were prepared with phosphate buffer solution (PBS, pH 7.0) and stored at 4 °C. The antibody was stored frozen, and standard solutions were prepared daily with PBS solution. All aqueous solutions were prepared in deionized, distilled water.

### 2.2. Equipment for a Batch-Type QCM Sensor

A QCM sensor purchased from Tamadevice Co., Ltd. (Tokyo, Japan) was used to estimate the amount of anti-CRP deposited. The frequency shift, Δf, of the quartz crystal after anti-CRP adsorption was measured. The frequency counter in the QCM was purchased from Turtle Industry Co., Ltd. (TUSB-S03CNBZ, Ibaragi, Japan). The resonance frequency was 30 MHz and the electrode diameter was 5.0 mm. The resonators were connected by silver conducting paste via wires to a BNC connector. The mass detection sensitivity of the QCM sensor is defined in terms of the mass of the detected target molecules per unit area of the gold surface for a given change in the resonance frequency of the QCM. The frequency was quantified with the mass change using the Sauerbrey equation. For the QCM sensor with a resonance frequency of 30 MHz, a frequency change of 1 Hz is estimated to be equivalent to 20 pg. The laboratory temperature was approximately 20 °C. A higher resonance frequency will lead to greater sensitivity of the QCM. The background signal is greatly amplified by external influences such as temperature fluctuations, vibrations, flow, and other factors.

The piezoelectric inkjet printing system used was manufactured by Cluster Technology Co., Ltd. (Osaka, Japan) [20,21]. The printing head (PulseInjector^®^) is a piezo-driven DOD head made from epoxy resin composite with a flat surface surrounding the ejection hole. By combining the PulseInjector^®^ and a dedicated driving unit (WaveBuilder^®^), it is possible to freely adjust the ejection drive waveform, the ejection rate, and the driving voltage, enabling droplets to be ejected on demand in picoliter quantities. The experimental system used for inkjet printing (DeskViewer^™^) can monitor the droplet trajectory and impact in real time for evaluating droplet ejection.

Anti-CRP aqueous solutions with concentrations from 0 to 1000 ng(x000B7)mL^−1^ were prepared using phosphate buffer solution (pH 7.4) and employed in this study. Carboxylic groups were introduced on the gold substrate of the QCM sensor with succinic anhydride, and the activated ester was prepared using *N*-ethyl-*N*-(3-dimethylaminopropyl) carbodiimide and *N*-hydroxysuccinimide. The substrate was reacted with the antibody to form amide bonds [22]. The PulseInjector^®^ had a 25-μm-diameter hole. The amount ejected per shot was estimated to be approximately 30 pL, judged from analysis of the droplet trajectories.

## 3. Results and Discussion

### 3.1. Background Noise Elimination Using Batch-Type QCM Sensor System

To reduce background noise from external sources such as air flow and temperature fluctuations, we applied a stainless-steel cover supplied with a commercially available QCM sensor. The resonance frequency of the QCM sensor was stable when the stainless-steel cover was securely fitted over the sensor and the temperature was constant.

**Figure 1.**
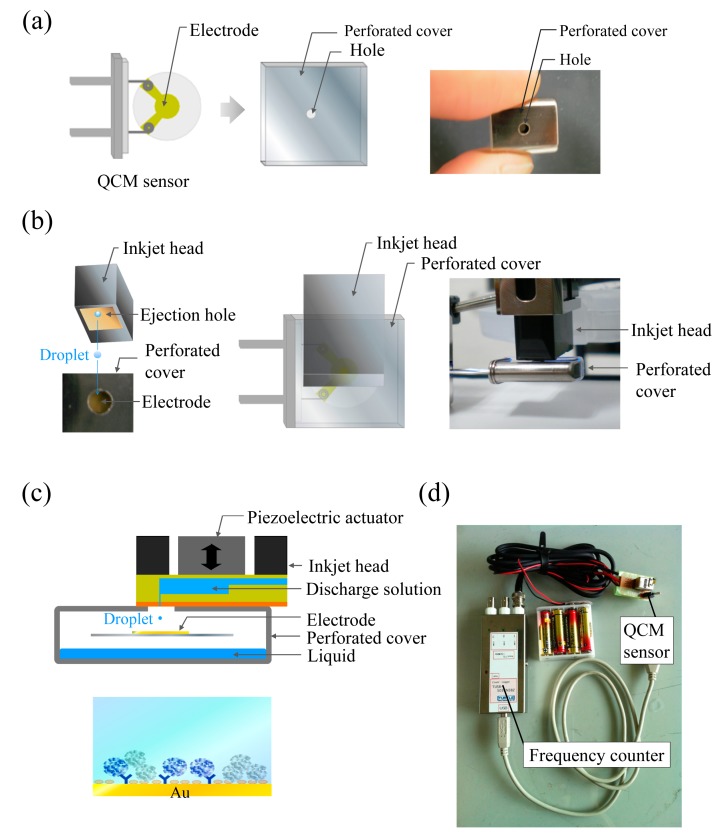
Experimental setup of a practical QCM sensor system using inkjet printing technology: (**a**) perforated stainless-steel cover; (**b**) the QCM sensor inserted into the cover and installation of the perforated cover on the inkjet head; (**c**) the hole of the cover is capped with the flat surface of the inkjet head, and an inkjet shot is deposited on the QCM sensor surface; (**d**) QCM sensor system using a commercially available device.

**Figure 2.**
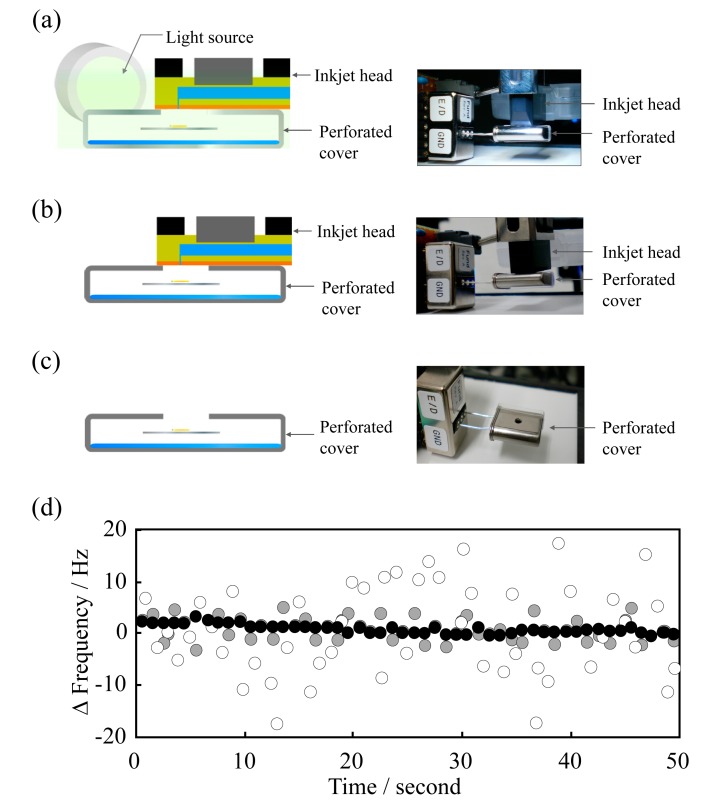
Elimination of external sources of background noise using the following methods: (**a**) perforated stainless-steel cover is capped with the flat surface of the inkjet head and light irradiation was supplied for a constant thermal energy (●); (**b**) perforated stainless-steel cover is capped with the flat surface of the inkjet head without light irradiation ( 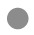); (**c**) perforated stainless-steel cover only (○); (**d**) QCM sensor frequency fluctuation.

The stainless-steel cover has a convenient shape for contact with the PulseInjector^®^. If a hole is made in the stainless-steel cover for droplet ejection and the cover closely contacts the PulseInjector^®^ (Figure 1a,b), it should be possible to reduce frequency fluctuations as efficiently as using the stainless-steel cover without a hole. Thus, a precisely-machined hole in the cover (Figure 1a,b) should allow droplet ejection while eliminating environmental fluctuations (Figure 1c). A simple QCM sensor unit, which is portable and inexpensive, was constructed using the perforated stainless-steel cover (Figure 1d). The frequency of a QCM sensor is influenced by external fluctuations in temperature. To minimize these fluctuations, the perforated stainless-steel cover was used as a measurement holder (Figure 2).

Since stainless-steel has high thermal conductivity, irradiation with light generates a constant flow of thermal energy and dissipates the thermal energy (Figure 2a). Furthermore, the cover can be securely fitted over the sensor and keeps the temperature constant during monitoring of the frequency shift of the QCM sensor. The light irradiation optical system was set 10 cm from the holder. In order to investigate the removal of the external fluctuations, a QCM sensor unit of different design was constructed, and the frequency changes were compared with (Figure 2a) and without (Figure 2b) light irradiation, and without installing the stainless-steel cover with no irradiation (Figure 2c).

As shown in Figure 2d, the results revealed that the frequency fluctuations were considerably different with the various setups. The average absolute values of the dispersion decreased from 33.1 to 8.71 Hz by installing the cover (Figure 2b,c). Furthermore, the light irradiation to the perforated cover decreased the dispersion more than in the absence of light, to 1.21 Hz.

Environmental temperature variations throughout the day and from day to day, even when using an air conditioner, are problematic due to the sensitivity of the QCM sensor. However, the relatively simple methods proposed here effectively minimized fluctuations in temperature.

### 3.2. Minimization of Evaporation in Measuring the Mass Change

**Figure 3.**
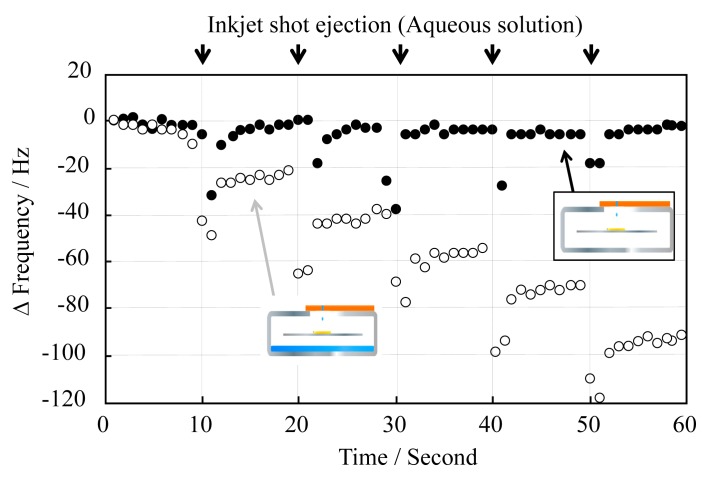
Variation in QCM frequency during 10-shot inkjet injections in a stainless-steel cover, with (○) and without (●) added water. Frequency changes were examined to evaluate the effect of the humidifying cover.

We next studied the minimization of evaporation, which prevents accurate detection of antigen-antibody interactions. The principle of detecting antigen-antibody interactions is based on a given change in the oscillation frequency generated by releasing the antigen from the electrode surface after deposition of the aqueous surfactant solution [phosphate buffer solution containing 0.1% (v/v) Triton X-100]. However, the frequency value returns to its original value right after an ejection due to evaporation. In order to prevent water from evaporating, we placed some water into the stainless-steel cover, and then sealed the hole with the flat surface of the PulseInjector^®^, thus creating a humidifying cover that can be completely sealed. Light irradiation generates heat intensity and produces a constant thermal energy, producing humid conditions under the cover. Thus, evaporation should have no direct effect on the determination of the frequency change (Figure 3).

Unlike when there was no water in the cover, the frequency varied in a step-like manner. When light was irradiated more than 10 min after sealing, loss by evaporation was no longer observed. This demonstrates that the QCM sensor unit can detect the interaction in an aqueous droplet deposited, free from the effects of evaporation loss.

### 3.3. Detection of Specific Binding in an Inkjet Deposition

**Figure 4.**
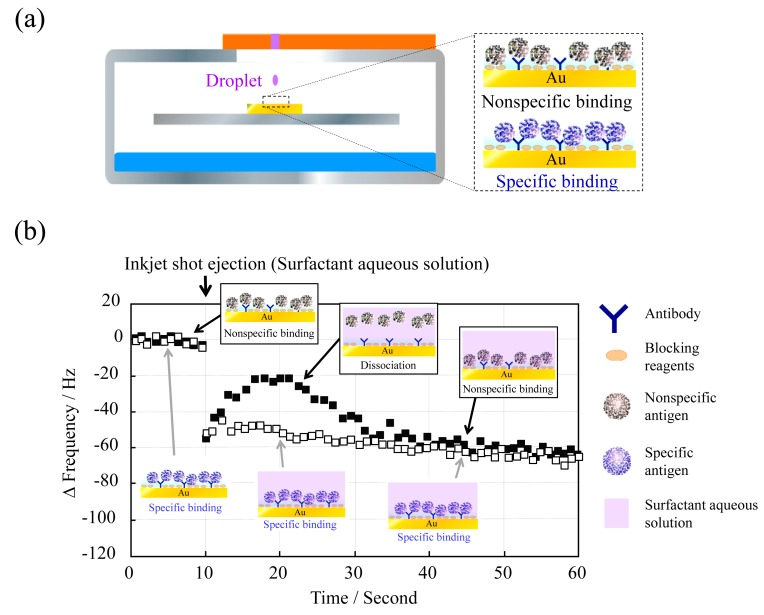
Discrimination of nonspecific binding from specific binding: (**a**) CRP and adiponectin were reacted with the anti-CRP immobilized QCM sensor; (**b**) QCM output obtained by 10-shot inkjet injections of the aqueous surfactant solution.

To investigate whether the QCM sensor unit can discriminate nonspecific binding from specific binding, we prepared the QCM sensor surface by reaction of either specific (CRP) or nonspecific (adiponectin) antigens with an anti-CRP-immobilized electrode (Figure 4a).

Aqueous surfactant solution, a phosphate buffer solution containing 0.1% (v/v) Triton X-100, was deposited at 10 s and then the frequency change as Δf versus time was measured (Figure 4b). The frequency change with adiponectin was considerably different from that with CRP, stemming from the release of adiponectin from the electrode surface. The frequency with adiponectin became almost the same after a short time (around 35 s) due to gravitational deposition. However, the frequency with CRP hardly changed after surfactant deposition at 10 s, since CRP was specifically bound to anti-CRP and was not released. To the best of our knowledge, there have been no reports in which specific and nonspecific binding have been distinguished in this manner.

**Figure 5.**
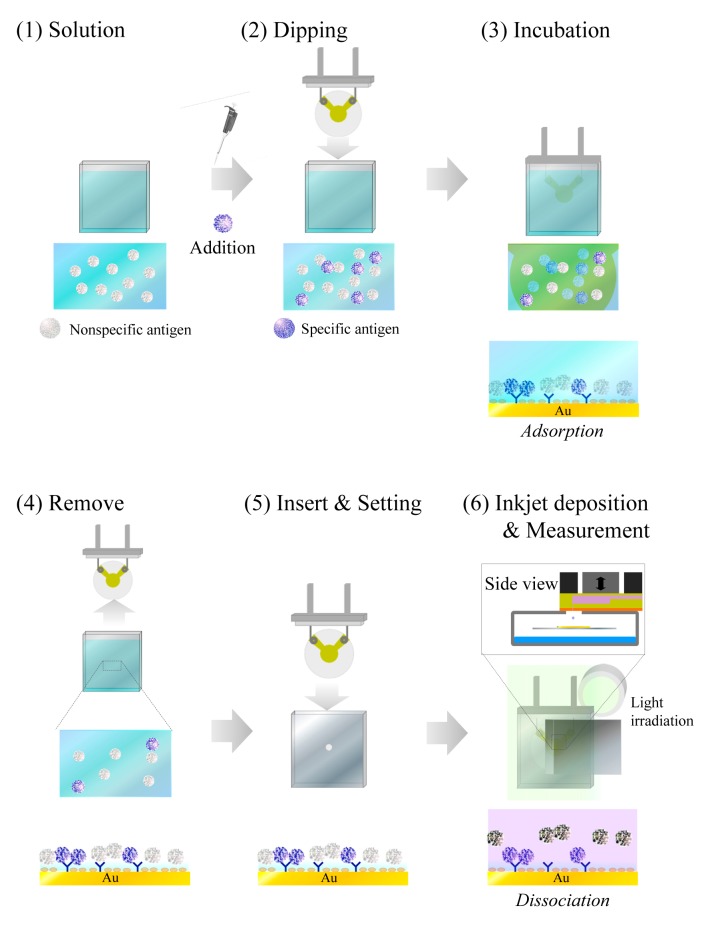
Immunoassay process with the developed QCM sensor unit.

For evaluation of the effectiveness of different concentrations of CRP, the QCM sensor surface was prepared by a commonly used conventional immunoassay batch-method process, as shown in Figure 5. This is based on a competitive immunoreaction between CRP and adiponectin and the incubation time was fixed at 1 h. Next, the QCM sensor was placed in the inkjet system, as described in Figures 2a and 3.

**Figure 6.**
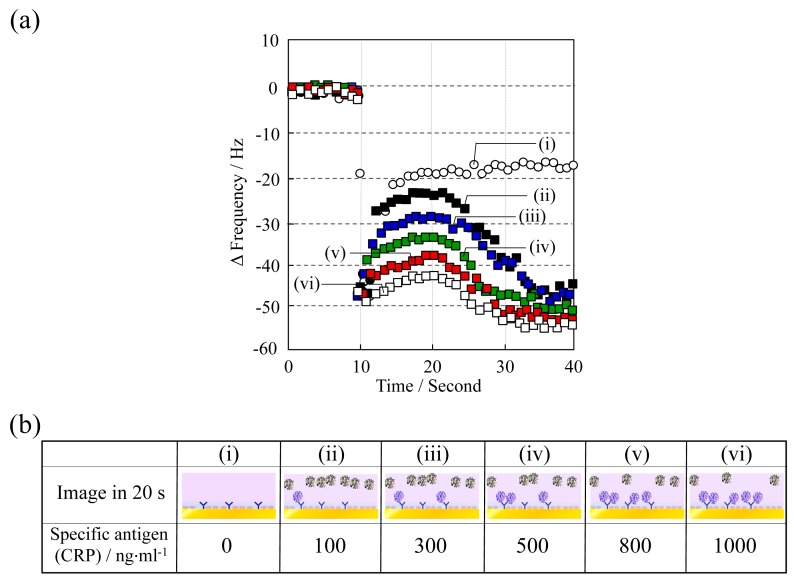
The frequency change as Δf *vs*. time at various CRP concentrations (0–1000 ng·mL^−1^): (**a**) QCM output obtained at different concentrations of CRP; (**b**) Adiponectin is released from the QCM sensor surface as the concentration of CRP increases.

**Figure 7.**
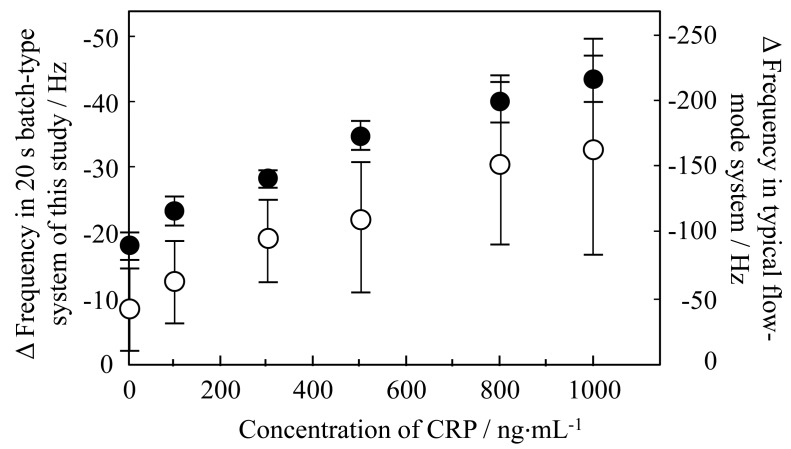
Analytical curve of frequency variation for different CRP concentrations from 0 to 1000 ng·mL^−1^: (**a**) batch-type system utilizing inkjet printing system (●); (**b**) typical flow-mode system (○). Three values of Δf at 20 s were averaged and plotted against sample concentration.

The frequency change as Δf *vs*. time at various CRP concentrations (0–1000 ng·mL^−1^) is shown in Figure 6. Aqueous surfactant solution was deposited at 10 s and adiponectin was employed at a concentration of 1000 ng·mL^−1^. As the CRP concentration increased, Δf after 10 s decreased and a linear shift was observed in the concentration range studied. The decrease in Δf stems from the reduction in the amount of adiponectin being released from the QCM sensor surface, because the concentration of adiponectin on the surface decreases in the competitive immunoreaction process as the concentration of CRP increases. Consequently, the magnitude of Δf at 20 s *vs*. CRP concentration was investigated (Figure 7). Additionally, the dependency and variations were compared with those of a typical flow-mode QCM sensor in order to illustrate the potential of this batch-type system. The anti-CRP immobilized QCM sensor was placed in the flow cell holder (ALS Co., Ltd., Tokyo, Japan), and then the solution containing the different concentrations of CRP antigen was injected by peristaltic pump onto the QCM sensor. Although good correlation was confirmed in this system, a large difference was observed in variation: the variation in the batch-type was considerably lower than in the typical flow-mode. This indicates that the batch-type system could offer a significant advantage in precise measurements.

The limit of detection was found to be 50 ng·mL^−1^ based on a signal-to-noise ratio of three, with a correlation coefficient of 0.957. High sensitivity CRP (hs-CRP) of <1000 ng·mL^−1^ is well known as a reliable biomarker in obesity, metabolic syndrome, and diabetes, thus it is critical to know the concentration level as a risk factor [23]. Therefore, good quantification in the range of 50–1000 ng·mL^−1^ allows for a meaningful result from the viewpoint of sensor devices in clinical diagnoses.

## 4. Conclusions

This study demonstrated a valuable approach to constructing a reliable batch-type QCM immunosensor by utilizing inkjet printing technologies and background noise removal techniques. The achieved sensor unit was constructed using only simple and commercially available devices. Three main advantages were achieved with the novel proposed device: (1) inkjet printing equipment was utilized as an alternative to an injection pump; (2) with basic commercial devices, it is possible to reduce the background noise to nearly zero; (3) the proposed QCM sensor can detect antigen-antibody interactions in an inkjet deposition. These achievements will lead to a reliable and practical QCM immunosensor system. Detailed research toward convenient and marketable QCM sensor technology with commercial potential will be performed and reported in the future.
